# Association of Inflammatory Sialoproteins, Lipid Peroxides and Serum Magnesium Levels with Cardiometabolic Risk Factors in Obese Children of South Indian Population

**Published:** 2014-06

**Authors:** G. Niranjan, D. Anitha, A. R. Srinivasan, V. Kuzhandai Velu, C. Venkatesh, M. Sathish Babu, R. Ramesh, S. Saha

**Affiliations:** 1 Department of Biochemistry, Mahatma Gandhi Medical College and Research Institute, Sri Balaji Vidyapeeth [SBV], Pillaiyarkuppam, Puducherry-607402, India;; 2 Department of Pediatrics, JIPMER, Puducherry-605006, India

**Keywords:** Protein Bound Sialic Acid, hsCRP, Insulin Resistance, Malondialdehyde, Serum Magnesium, Childhood Obesity, Type 2 Diabetes Mellitus

## Abstract

The Incidence of childhood obesity and metabolic syndrome is increasing even in rural and semi-urban regions of India. Adipose tissue mass secretes several inflammatory proteins, which could potentially alter the metabolic processes, leading to several complications at the later stages of life. With limited studies on protein bound sialic acid (PBSA) as a marker of oxidative stress mediated inflammation in obese children, this study was aimed to assess and correlate PBSA with lipid peroxidation and other cardiometabolic risk factors like Insulin Resistance (IR), serum magnesium, and high sensitive C reactive Protein (hsCRP) levels in order to provide an insight into the degree of systemic inflammation and oxidative stress. This study included 62 obese children (≥95% percentile of the *CDC chart*) and 60 non obese controls. This study documents significant higher levels of PBSA, IR, Malondialdehyde (MDA), hsCRP and uric acid in obese children (*p*<0.001). PBSA was associated with IR, hsCRP, uric acid, hypomagnesaemia. Higher degrees of oxidative stress, Insulin resistance and low serum magnesium levels were noted in obese children. PBSA and hsCRP levels were elevated and were associated with Insulin resistance in obese children of South Indian population.

## INTRODUCTION

Childhood obesity in recent years has become a serious public health problem worldwide (1). Several studies have documented the increasing incidence of childhood obesity even among rural and semi-urban populations (2). Sedentary lifestyle habits, preference for indoor games, academic stress of modern day schools and westernization of diet are some of the factors which have led to the increased burden of obesity in children of school going age (3). Screening and providing health education for children is essential to prevent the future complications such as metabolic syndrome, Type 2 Diabetes Mellitus (T2DM) and cardiovascular diseases in the later ages (4). These complications of obesity are attributed to the various inflammatory sialoproteins which are secreted from the adipocytes mass (5). Although Body Mass Index (BMI), Waist Circumference (WC) and Waist-Hip Ratio (WHR) are frequently being used as the anthropometric indices of overweight and obesity in adults, there are only few studies on Indian populations, as related to the efficacy of such anthropometric measures in children (6-8). Central adiposity, as represented by increased WC is more active and hence dangerous when compared to peripheral obesity (9).

Obesity, especially abdominal (central) is associated with oxidative stress (10). Oxidative stress in association with systemic inflammation affects both insulin secretion and its action, thus resulting in poor glycemic control (11, 12). In addition, there exist an influence of various inflammatory sialoproteins secreted from the adipocyte mass on Insulin Resistance (IR), serum lipids & glycemic control (13-15).

The biomolecules *in vivo* including the plasma proteins undergo several modifications upon exposure to Stress. Commonly observed among such modifications is glycosylation. Proteins undergo glycosylation with the terminal sialic acid residues under stress (16). It is interesting to note that several such inflammatory proteins produced in obesity are glycoproteins (16). High sensitive C-reactive protein (hsCRP) is an acute phase protein elevated in various inflammatory conditions (17-19). However, significance of hsCRP and other sialoproteins and their association with the metabolic risk factors in Indian children is yet to be established unambiguously. Estimation of Protein Bound Sialic Acid [PBSA] and hsCRP could thus provide an idea regarding the degree of systemic inflammation and oxidative stress in obese children. Studies evaluating the efficacy of these markers as the diagnostic and prognostic indicators in childhood obesity are sparse.

Magnesium is a vital divalent metal ion and a cofactor for several enzymes involved in the metabolism of carbohydrates and also assists the action of insulin (20). Studies have shown the association of hypomagnesaemia with oxidative stress (21) but, still we do not have supportive evidences to apprise us of the fact whether it is the cause or the effect. Uric acid levels in serum are known to be higher in patients with obesity and metabolic syndrome (22). This study was planned in the light of above mentioned scientific information and also considering the lack of objective studies from South India related to childhood obesity. It is also to be noted that there is a rising burden of childhood obesity, a pronounced morbidity in Indian population (1, 2).

## METHODOLOGY

In this cross-sectional study, we included sixty two obese children who were ≥95% percentile of the CDC *(Centre for Disease Control and Prevention, 2000)* chart (23) and sixty age and gender matched controls who had met our selection criteria, following the written informed consent from their parent/guardian. An assent was also taken from the children willing to participate in this study. The ethical clearance for conducting this study was obtained from Institutional Human Ethical Committee, MGMC & RI. Children with Diabetes Mellitus (Type1 or Type 2), autoimmune disorders, nephrotic syndrome and any other endocrinal disorders were excluded from this study. Children with any signs of acute/chronic infections or inflammation were not recruited. Blood pressure and the anthropometric measures were recorded. With three ml of venous blood samples, the biochemical parameters were analyzed. Fasting Plasma Glucose (FPG), fasting plasma insulin, HbA_1c_, urea, creatinine, magnesium and serum lipids were estimated by International Federation of Clinical Chemistry and laboratory Medicine approved methods. PBSA was estimated by *Aminoff’s method* (24). Homeostasis Model Assessment for Insulin Resistance [HOMA-IR] was calculated by using the formula HOMA-IR= [FPG X FPI/405]. MDA was estimated by using *OxiSelect^TM^ TBARS* Assay Kit. Data were expressed as mean ± SD; unpaired student t-test was used to compare the data. A p value <0.05 was considered as the level of significance for all statistical purposes. SPSS version: 19 for Windows was used for all statistical analyses (SPSS Inc., Chicago, USA). Internal quality control was effected through the control samples (sera) provided by M/S Biorad USA. EQAS was provided by the Christian Medical College (CMC), Vellore, India.

## OBSERVATIONS AND RESULTS

All the observations were recorded and expressed as means ± SD. Age, gender, anthropometric measures of the obese and non-obese children were tabulated and compared using unpaired Student’s *‘t’* test (Table 1). The age and gender distribution was uniform among the study participants of both the groups. All the anthropometric measures *viz.,* BMI, WC, and WHR were significantly high in obese children. The differences between average systolic and diastolic blood pressures were not significant between the groups.

The serum lipids were not significantly different in cases when compared to the control group with the exception of triacylglycerols (Table 1).

**Table 1:** Comparison of physiological, anthropometric parameters and serum lipid levels between obese and non-obese children

Parameters	Controls (n=60)	Cases (n=62)	‘*p*’ Value

Age (years)	9.81 ± 1.5	9.34 ± 1.6	0.09
Gender (M/F)	M=36%, F=64%	M=38%, F=62%	
Weight (Kg)	42.6 ± 7.33	57.74 ± 6.84	<0.001^a^
Height (cm)	145.2 ± 7.3	144.2 ± 7.9	0.17
BMI (Kg/m^2^)	15.55 ± 2.55	20.89 ± 2.41	<0.001^a^
WC (cms)	51.75 ± 2.81	58.24 ± 2.7	<0.001^a^
WHR (cm)	29.25 ± 2.79	32.84 ± 2.90	0.002a
SBP (mmHg)	118.31 ± 3.16	117.11 ± 1.21	0.91
DBP (mmHg)	80.50 ± 2.31	79.31 ± 3.10	0.86
TC (mg/dl)	155.01 ± 30.58	159.93 ± 32.07	0.42
TAGs (mg/dl)	102 ± 57.53	117.09 ± 65.55	0.01^a^
LDL-C (mg/dl)	84.94 ± 21.06	86.35 ± 27.74	0.90
HDL-C (mg/dl)	47.34 ± 10.16	46.06 ± 12.14	0.55
VLDL (mg/dl)	25.74 ± 27.06	27.19 ± 16.33	0.73

a Indicates the p value is statistically significant. WC, Waist Circumference; WHR, Waist Hip Ratio; BMI, Body Mass Index; SBP, Systolic Blood Pressure; DBP, Diastolic Blood Pressure; TC, Total Cholesterol; TAGs, Tri Acyl Glycerols; LDL-C, Low Density Lipoprotein - Cholesterol; HDL-C, High Density Lipoprotein-Cholesterol; VLDL, Very Low Density Lipoproteins.

The comparison of inflammatory, oxidative stress, IR, glycemic control and renal function parameters are depicted in Table 2. We observed significantly high plasma insulin, PBSA, MDA, uric acid and hsCRP levels in obese children compared to the non-obese children. Hypomagnesaemia was seen in obese children (Table 2). The mean FPG and HbA_1c_, urea, creatinine and total protein concentrations were not significantly different between the two groups.

**Table 2:** Comparison of Biochemical parameters between obese and non-obese children

Parameters	Controls (n=60)	Cases (n=62)	‘*p*’ Value

FPG (mg/dl)	88.67 ± 7.39	90.11 ± 13.81	0.24
HbA_1c_ (%)	5.58 ± 0.53	5.71 ± 0.33	0.49
FPI (μU/ml)	6.59 ± 0.74	8.30 ± 0.62	0.002^a^
HOMA-IR	1.44 ± 0.78	1.84 ± 0.92	0.003^a^
Serum Magnesium (mg/dl)	3.31 ± 0.49	2.03 ± 0.33	<0.001^a^
PBSA (μgm/mg of proteins)	2.45 ± 0.36	3.79 ± 0.78	<0.001^a^
Serum MDA (μmol/l)	6.30 ± 1.13	8.81 ± 1.02	<0.001^a^
Serum hsCRP (mg/l)	0.46 ± 0.27	0.87 ± 0.47	0.001^a^
Serum Urea (mg/dl)	24.32 ± 1.53	24.55 ± 1	0.45
Serum Creatinine (mg/dl)	0.94 ± 0.08	1.01 ± 0.22	0.08
Serum Uric acid (mg/dl)	5.16 ± 1.46	6.11 ± 0.73	0.002^a^
Serum Total protein (gm/dl)	6.28 ± 0.42	6.33 ± 0.41	0.23

a Indicates that the values are statistically significant. FPG, Fasting Plasma Glucose; FPI, Fasting Plasma Insulin; HOMA-IR, homeostasis model assessment for Insulin Resistance [FPG X FPI /405]; PBSA, Protein Bound Sialic Acid; MDA , Malondialdehyde; hsCRP, high sensitive C-Reactive Protein.

The association of HbA_1c_ with the anthropometric measures was evaluated. *Pearson’s* correlation analysis for the parametric data was used to find out the association between HbA_1c_ and anthropometric measurements. The difference of association ie., p and r values between cases and controls were also evaluated by using student’s ‘*t*’ tests. WC, WHR, BMI were significantly associated with HbA_1c_. This association was significantly high in cases compared to controls for WC (*p*<0.005). Table 3 shows the association of IR with PBSA, hsCRP, HbA_1c_, magnesium and uric acid levels. The association was statistically significant among IR and other cardiometabolic risk factors in obese children.

**Table 3:** Association of Insulin Resistance with hsCRP, PBSA, magnesium, HbA_1c_ and uric acid levels between obese and non-obese children

Parameters	Cases (n=62)		Controls (n=60)		‘*p*’ Value
	r value	p value	r value	p value	

IR v/s PBSA (μgm/mg of proteins)	0.424	0.003^a^	0.077	0.558	0.042^a^
IR v/s hsCRP (mg/L)	0.716	0.00^a^	0.133	0.31	0.002^a^
IR v/s HbA_1c_ (%)	0.219	0.093	0.036	0.803	0.192
IR v/s magnesium (mg/dl)	0.588	0.000^a^	0.012	0.927	0.001^a^
IR v/s Uric acid (mg/dl)	0.367	0.004^a^	0.017	0.906	0.043^a^

a Indicates a *p* value of <0.05, which is statistically significant. IR, Insulin Resistance; PBSA, Protein Bound Sialic Acid; hsCRP, high sensitive C Reactive Protein.

## DISCUSSION

In this study, the various anthropometric, physiological and the biochemical variables were evaluated in obese children and compared with controls. The age and the gender distribution of study participants were uniform. BMI, WHR and WC were significantly higher in obese children, irrespective of gender (Table 1). The structure and functions of biomolecules get altered upon exposure to free radicals. MDA, a marker of free radical induced damage to lipids and is elevated in several conditions with oxidative stress (25). In this study, MDA levels were significantly higher in obese children indicating higher degree of oxidative stress in them (Table 2). Obesity & T2DM are associated with oxidative stress due to flux of excess glucose into the polyol pathway which is the root cause of IR leading to the micro and macrovascular complications (12).

Although it is not proven as yet whether it is the cause or the effect, hypomagnesaemia is also known to be associated with higher degree of oxidative stress (26). Studies on different population have also documented high levels of serum MDA (27, 28), and low levels of serum magnesium in obese children (29). Lack of nutritious diet, westernization of dietary habits, osmotic diuresis in obesity and T2DM and also the lack of physical activity in modern day children are certain factors that contribute to hypomagnesaemia.

The fat deposited in the central part of the body as indicated by increased WC (central obesity), is more active and dangerous in comparison to the fat distributed in the peripheral parts (9). There are several studies in adults documenting higher levels of adipokines and other inflammatory proteins in obesity (5). hsCRP, although not specific to any inflammatory condition as such, is a sensitive and reliable marker of systemic inflammation, in general (17-19). Moreover, many inflammatory proteins are glycoproteins with sialic acid as terminal carbohydrate moiety. Hence, estimation of PBSA levels is an objective and reliable measure to assess systemic inflammation as well as oxidative stress. In the current study, significantly high levels of PBSA and hsCRP in obese children were seen (Table 2). Uric acid, a known marker of metabolic syndrome was noted to be higher in obese children in comparison to the non-obese children (Table 2).

Pearson’s correlation was drawn to find out the association among the various physiological, biochemical and anthropometric parameters in children with obesity. We observed a significant positive correlation between IR and hsCRP. IR was also significantly associated with uric acid hypomagnesaemia and PBSA levels. WC, irrespective of the gender in children proved to be a better and sensitive anthropometric screening tool in preference to WHR and BMI. We did not find any significant difference in serum lipids, systolic or diastolic blood pressure.

This study unmasks the ice-berg phenomenon with obesity at its tip. Higher degrees of systemic inflammation and oxidative stress are the potential cardiometabolic risk factors which need to be necessarily addressed. Obese children possess higher risk of developing T2DM, hypertension and other metabolic diseases in the later ages of their lives. This calls for the importance of screening obese children and intervening at the primordial phase. The interventional methods include health education, supplementation of micronutrients especially magnesium and physical exercise. The intensity of systemic inflammation & oxidative stress in obese children is similar to that of adults. Intervention in these children should be initiated at the earliest. This needs an aggressive inter-disciplinary approach including counseling (28). This study should adequately forewarn the health professionals and policymakers to consider childhood obesity as an important issue that needs to be addressed especially at a juncture where there is a growing increase in the incidence of T2DM. Moreover, this initiative also would benefit their parents/guardians.

## CONCLUSION

Higher levels of MDA, Insulin resistance and hypomagnesaemia were documented in obese children of South Indian population. Insulin resistance in obese children was associated with high levels of inflammatory sialoproteins.
